# The Effects of Velvet Antler of Deer on Cardiac Functions of Rats with Heart Failure following Myocardial Infarction

**DOI:** 10.1155/2012/825056

**Published:** 2012-04-24

**Authors:** Ming-Jing Shao, Shuo-Ren Wang, Ming-Jing Zhao, Xi-Ling Lv, Hao Xu, Lin Li, Huan Gu, Jiu-Liang Zhang, Ge Li, Xiang-Ning Cui, Li Huang

**Affiliations:** ^1^National Integrated Traditional and Western Medicine Center for Cardiovascular Disease, China-Japan Friendship Hospital, Beijing 100029, China; ^2^Laboratory of Traditional Chinese Internal Medicine, Dongzhimen Hospital, Beijing University of Chinese Medicine, Beijing 100700, China

## Abstract

Velvet antler of deer (VAD) is a commonly-used kidney-Yang supplementing traditional Chinese medication. According to the heart-kidney-related theory, heart Yang originates in kidney Yang and heart failure due to heart Yang deficiency can be treated by tonifying kidney Yang. In this study, we investigated therapeutic effects of VAD on cardiac functions in rats with heart failure following myocardial infarction. Forty-eight male Wistar rats were subjected either to left coronary artery ligation (*N* = 36) or to sham operation (*N* = 12). One week after the surgery, rats with heart failure received daily treatment of double-distilled water, captopril or VAD by gavage for consecutively four weeks, while sham-operated animals were given double-distilled water. Ultrasonic echocardiography was adopted to examine cardiac structural and functional parameters and serum brain natriuretic peptide (BNP) concentration was measured using radioimmunoassay. We found that VAD partially reversed changes in cardiac functional parameters and serum BNP levels in rats with heart failure. These results provide further evidence for the heart-kidney-related theory and suggest that VAD might be a potentially alternative and complementary medicine for the treatment of heart failure.

## 1. Introduction

Velvet antler of deer (VAD) is a precious traditional Chinese medication, warm in nature and sweet and salty in flavor, and is commonly used to treat various diseases by supplementing kidney Yang. According to TCM theories, kidney Yang deficiency can be presented in the symptoms including declining libido, soreness, or cold sensation in the knees and lumbar regions, spiritual fatigue, and so forth. Both clinical and animal studies have shown that VAD can promote the development of reproduction systems, relieve the pain of arthritis, nourish the neural cells, and so on [1]. These pharmacological effects provide empirical evidence for its role of tonifying kidney Yang. 

Heart failure (HF) is a clinical syndrome which all types of heart diseases will eventually develop into. Evidence from epidemiological studies has demonstrated that the incidence of HF in Chinese adults was 0.9%: it reached 0.7% in males and 1.0% in females [2]. From the perspective of the traditional chinese medicine (TCM), the primary cause of HF is heart Yang deficiency that results from Qi inadequacy and blood stasis. According to the heart-kidney-related theory of TCM, heart Yang originates in kidney Yang, and therefore it is hypothesized that tonifying kidney Yang could be used to strengthen heart Yang and treat HF. Indeed, VAD has been reported to have protective effects on the damaged heart muscle cells in the animal models of myocardial infarction via various mechanisms, such as reducing the release of endothelin [3], promoting superoxide dismutase activities, and decreasing serum malondialdehyde levels [4], and increasing the levels of nitric oxide and calcitonin-gene-related peptide [5]. In addition, oral administration of VAD has been found to strengthen the pulse, increase blood pressure, and enhance heart sounds for chronic circulatory disorders accompanied by hypotension [6]. However, to the best of our knowledge, the therapeutic effects of VAD on the heart failure have not been fully elucidated due to a lack of clinical and animal studies.

Left coronary artery ligation is one of the widely used animal models to mimic myocardial infarction and heart failure in patients [7]. Echocardiography and serum brain natriuretic peptide (BNP) levels are two commonly used indices to evaluate and diagnose heart failure [8]. To provide empirical evidence for VAD's clinical application in the treatment of heart diseases, in the current study, we investigated its therapeutic effects on heart failure following myocardial infarction with captopril as the positive control by evaluating echocardiographic parameters and serum BNP levels.

## 2. Methods and Materials

### 2.1. Animals and the Heart Failure Model

Male Wistar rats (*N* = 48, age 8 weeks, weighing 230 ± 20 g) were purchased from the Institute of Laboratory Animal Science, Chinese Academy of Medical Sciences, Beijing, China. They were housed four/cage in a controlled environment (23 ± 1°C; 45%–50% relative humidity; fixed 12/12 h light/dark cycle, lights on at 08:00 h) with food and water ad libitum. All procedures were performed in accordance with the National Institute of Health's Guide for the Use and Care of Laboratory Animals and were approved by the Committee on Animal Care and Use of the China-Japan Friendship Hospital.

After seven-day acclimation, animals had their body weights measured at first and then were anesthetized with intraperitoneal injection of 1% pentobarbital sodium. They were fixed on their backs, intubated using a 16-gauge catheter, and artificially ventilated (80 stokes/min, 0.4 L/250 g) with a pressure-controlled respirator for small animals (RSP1002, Kent Scientific Corporation, CT, USA). An incision of the skin and intercostal muscles was made between the third and fourth ribs. Under the monitor of a lead 2 electrocardiogram (XJJ-11, Kent Scientific Corporation, CT, USA), a thoracotomy was performed and the pericardium was opened, which left the heart adequately exposed. To induce myocardial infarction, the left coronary artery was ligated 1.5–2 mm from the aortic root between the pulmonary cone and the left auricular appendage with silk sutures. Then the muscles and skins were sutured and the ventilation was stopped when rats' heart rate and respiration went steady. The sham-operated animals underwent the same procedure except that the silk suture was placed around the left coronary artery without being tied. After the surgery, all animals were injected with penicillin for three days to prevent infection. Six rats with induced myocardial infarction died during the operation whereas two rats in the sham group died of infection. The operative mortality was 16.7%.

### 2.2. Drugs and Pharmacological Procedures

 The powder of velvet antler of deer (VAD, The Scientific and Technological Development Center of Qingyuan Manchu Autonomous County, Liaoning Province, China) and captopril (CAP, Beijing Shuguang Pharmaceutical Co., Ltd., Beijing, China) were triturated and then well suspended in double-distilled water at the doses of 20 mg/mL and 1 mg/mL respectively. These doses were calculated according to the conversion table of animal doses to human equivalent doses based on body surface area [9]. Provided that the doses of rats should be seven times lower than those of humans, the clinical dose of VAD was 2 g/70 Kg and hence the equivalent dose for rats is 200 mg/Kg. Similarly, the clinical dose of captopril is 100 mg/70 Kg, and for rats the dose should be 10 mg/Kg. One week after the surgery, animals with myocardial infarction following left coronary artery ligation received daily treatment of double-distilled water (referred as the HF group), captopril (the HF + CAP group), or velvet (the HF + VAD group) by gavage (1 mL/100 mg body weight) for consecutively four weeks, while sham-operated animals (the SHAM group) were administered double-distilled water once daily for four weeks.

### 2.3. Measurement of Cardiac Structural and Functional Parameters

 Echocardiography (HDI 5000, Philips) was performed before rats were sacrificed. Under anesthesia by intraperitoneal injection of 40 mg/kg pentobarbital sodium, rats were fixed on their backs with their fur shaved and skin cleaned. Using a high-frequency linear-array transducer (CL15-7), the structural parameters of the heart included left atrial diameter (LAD), left ventricular end-diastolic diameter (LVDd), left ventricular end-systolic diameter (LVDs), end-systolic interventricular septal thickness (IVSTs), end-diastolic interventricular septal thickness (IVSTd), left ventricular end-systolic posterior wall thickness (LVPWTs), and left ventricular end-diastolic posterior wall thickness (LVPWTd). Besides, two functional parameters were calculated by the following formulas: left ventricular short-axis fractional shortening (LVFS) = (LVDd-LVDs)/LVDd, left ventricular ejection fraction (LVEF) = (LVDd^3^- LVDs^3^)/ LVDd^3^.

### 2.4. Brain Natriuretic Peptide (BNP) Measurement

Blood samples were collected in EDTA tubes, which was then placed on ice and centrifuged within 30 min at 4°C. The plasma was stored at −80°C until assay. Radioimmunoassays for rat BNP were performed as previously reported [10]. Briefly, standards or samples were incubated with antibody for rat BNP for 24 hours at 4°C. ^125^I-BNP (10 000 cpm) was then added, followed by additional incubation for 24 hours at 4°C. Then after 90 min of incubation with a secondary antibody, free and bound fractions were separated, and the radioactivity of the bound fraction was measured by a gamma counter (EG&G Wallac, USA).

### 2.5. Statistical Analysis

All data were expressed as means ± standard deviation. Group differences were evaluated using one-way analysis of variance (ANOVA). If the variance of the data was heterogeneous, the Kruskal-Wallis test was adopted, which was followed by the Nemenyi multiple comparison test. *P* < 0.05 was considered to be statistically significant.

## 3. Results

### 3.1. Effects of VAD and CAP on the Cardiac Structural Parameters in Rats with Heart Failure following Myocardial Infarction (Table 1)

 Compared to the SHAM group, the diameters of left atrium and left ventricle (LAD, LVDd, and LVDs) of animals in the heart failure groups were significantly increased, the interventricular septal thickness (IVSTd and IVSTs) was decreased (*P* < 0.001) whereas no group differences were found with respect to LVPWTd and LVPWTs (*P* > 0.05). VAD and CAP treatment did fail to reverse the effects of myocardial infarction on cardiac structural parameters (*P* > 0.05).

### 3.2. Effects of VAD and CAP on the Cardiac Functional Parameters in Rats with Heart Failure following Myocardial Infarction (Table 2)

 Compared to the SHAM group, animals in the heart failure groups had significantly lower LVFS and LVEF (*P* < 0.001), indicating that cardiac function was severely impaired by myocardial infarction. Further comparisons with the heart failure groups showed that the LVFS of the VAD group was significantly elevated than that of the HF group (*P* < 0.05) whereas the similar trend in the CAP group did not reach significance (*P* = 0.377). The LVEFs of both treatment groups were higher than that of the AMI group (*P*s < 0.05 for the VAD group and the CAP group). These results indicated that both VAD and CAP partially reversed the functional damage induced by myocardial infarction. No differences were found between the two treatment groups (*P* > 0.05).

### 3.3. Effects of VAD and CAP on Serum BNP Levels in Rats with Heart Failure following Myocardial Infarction (Table 3)

The serum BNP levels of the HF group were significantly higher than the sham group (*P* < 0.001), a change that was reversed by both VAD and CAP treatments (*P* < 0.05). No differences were found between the two treatment groups (*P* > 0.05).

## 4. Discussion

Although there is no such a term as “heart failure” (HF) in traditional Chinese medicine (TCM), its symptoms can be classified into the categories of dyspnea, palpitation, and edema. This disease is a condition of heart Qi deficiency and heart Yang devitalization due to Zang-Fu dysfunction that could be resulted from various factors such as improper diet, overstrain, and repeated invasion of exterior pathogens. The heart belongs to fire, governs blood and vessels, and controls spirit. It is a vital organ of the body just like a monarch to a country and plays a leading role in physical activities. The kidney belongs to water and is the organ which stores essence as well as true Yin and true Yang. It is the congenital origin and the root of life. Heart Yang originates in kidney Yang. Normal physical activities of the human body must rely on mutual coordination and constraints of these two organs. The outflow of heart Yang is the primary force that warms kidney Yang. The deficiency of Yang in the heart and kidney leads to weak contraction, which causes vascular obstruction and blood stasis, and hence the symptoms of heart failure appear. Just as *plain questions: Treatise on disharmony* said, “People who cannot lie down because that will make them gasp and cough are those who have too much water in their body. Water generates from fluid. Kidney is an organ of water, governing fluid flows and therefore controls lying down and gasping as well.” Accordingly, deficiency of the heart- and kidney Yang is the internal causes of heart failure and plays the pivotal role in the pathogenesis and prognosis of the disease.

Previous studies have shown that the TCM syndrome-type of rats with heart failure following myocardial infarction in the current study may belong to the syndrome of deficiency of Qi and blood stasis [11]. The pathogenesis mechanisms could be that coronary artery ligation caused blood stasis in the heart, which leads to Qi damage and produces symptoms of Qi deficiency in the organ and the whole body. Treatment that promotes blood circulation and replenishes Qi could improve cardiac function. Considering that Qi deficiency could develop into Yang deficiency and that kidney Yang is the origin of heart Yang, it is hypothesized that tonifying kidney Yang would strengthen heart Yang, which further supplements heart Qi and makes the heart beat stronger. These effects could be beneficial for the treatment of heart failure.

Based on the heart-kidney-related theory of TCM, in this study we investigated the therapeutic effects of VAD, a precious kidney Yang supplementing medicine, on cardiac structure and function as measured in echocardiography and serum BNP levels in rats with HF induced by myocardial infarction. BNP is a 32-amino-acid polypeptide secreted by the ventricles of the heart in response to excessive stretching of heart muscle cells and therefore has been used as an objective index reflecting the immediate condition of cardiac function and clinical severity of HF [8]. The finding that VAD treatment elevated LVFS/LVEF and decreased BNP levels provides evidence for the protective effects of VAD for the damaged heart. Recent studies have implicated multiple mechanisms of VAD in improving cardiac function. For instance, VAD could reduce the release of endothelin, prevent vascular contraction in the ischemic regions and enhance local coronary circulation [3]. VAD has also been reported to play a protective role on secondary injury of ischemic myocardium by promoting superoxide dismutase activities and decreasing serum malondialdehyde levels [4]. Lastly, VAD could elevate the levels of nitric oxide and calcitonin-gene-related peptide so as to modulate cardiomyocyte apoptosis [5].

We did not observe the therapeutic effects of VAD in cardiac structural parameters, a finding in contrast to a study showing that chronic treatment with VAD could significantly reverse the enlargement of left ventricle caused by myocardial infarction [12]. The inconsistency may be due to that our study used VAD powder whereas the latter study used the extracts of VAD, which contains a higher proportion of active components. Our finding that four-week treatment of VAD showed differential therapeutic effects on cardiac structure and function suggests that the treatment period of four weeks may be not long enough to induce structural changes of the heart as measured by echocardiography. According to our observation, VAD treatment can also reverse myocardial fibrosis of HF (unpublished data), which may serve as the structural basis of functional changes induced by VAD.

In the current study, we used captopril as a positive control and found that VAD and captopril showed comparable effects in reversing changes in functional parameters and BNP levels in rats with heart failure. Although VAD is more expensive than captopril, it has edges over captopril for patients with heart failure accompanied with lower blood pressure given the hypotensive side effect of captopril.

In conclusion, the kidney Yang supplementing drug, VAD, shows comparable therapeutic effects with captopril for heart failure induced by myocardial infarction as revealed by functional parameter changes in echocardiography and serum BNP levels. These results provide evidence for the heart-kidney-related theory of TCM and demonstrate that heart failure due to heart Yang deficiency can be treated by strengthening kidney Yang. Therefore, VAD might be a potentially alternative and complementary medicine used in the treatment of heart failure.

## Figures and Tables

**Table 1:** Comparison of cardiac structural parameters as revealed by echocardiography in rats ($\overset{̅}{x}\pm s$).

Group	*N*	LAD (mm)	IVSTd (mm)	IVSTs (mm)	LVPWTd (mm)	LVPWTs (mm)	LVDd (mm)	LVDs (mm)
sham	10	3.31 ± 0.15	1.22 ± 0.19	1.36 ± 0.20	2.13 ± 0.33	2.67 ± 0.33	5.79 ± 0.74	4.21 ± 0.64
HF	9	4.22 ± 0.23***	0.68 ± 0.14***	0.90 ± 0.17***	2.03 ± 0.32	2.58 ± 0.30	9.79 ± 0.66***	8.91 ± 0.71***
HF + CAP	11	4.42 ± 0.22***	0.66 ± 0.14***	0.91 ± 0.13***	1.88 ± 0.23	2.44 ± 0.27	10.28 ± 0.69***	9.18 ± 0.64***
HF + VAD	10	4.31 ± 0.30***	0.72 ± 0.13***	0.96 ± 0.12***	2.87 ± 0.33	2.48 ± 0.3	9.91 ± 0.71***	8.59 ± 0.63***

Notes: ***compared to the sham group, *P* < 0.001; HF = heart failure; CAP = captopril; VAD = velvet antler of deer.

**Table 2:** Comparison of functional parameters as revealed by echocardiography in rats ($\overset{̅}{x}\pm s$).

Group	*N*	LVFS (%)	LVEF (%)
sham	10	27.20 ± 6.16	60.60 ± 9.01
HF	9	9.11 ± 2.62***	24.67 ± 6.44***
HF + CAP	11	10.64 ± 2.11	32.82 ± 5.04Δ
HF + VAD	10	13.40 ± 2.91Δ	34.80 ± 6.84Δ

Notes: ***compared to the sham group, *P* < 0.001; Δ compared to the AMI group, *P* < 0.05. HF = heart failure; CAP = captopril; VAD = velvet antler of deer.

**Table 3:** Serum BNP levels of all groups ($\overset{̅}{x}\pm s$).

Group	*N*	BNP (pg/mL)
sham	10	0.89 ± 0.21
HF	9	1.31 ± 0.21***
HF + CAP	11	1.08 ± 0.16 Δ
HF + VAD	10	1.10 ± 0.18 Δ

Notes: *** compared to the sham group, *P* < 0.001; Δ compared to the AMI group, *P* < 0.05. HF = heart failure; CAP = captopril; VAD = velvet antler of deer.
